# Analysing The Cross-Section of The Abdominal Aortic Aneurysm Neck and Its Effects on Stent Deployment

**DOI:** 10.1038/s41598-020-61578-y

**Published:** 2020-03-13

**Authors:** Faidon Kyriakou, William Dempster, David Nash

**Affiliations:** Department of Mechanical and Aerospace Engineering, University of Strathclyde, 75 Montrose Street, Glasgow, G1 1XJ UK

**Keywords:** Anatomy, Interventional cardiology, Biomedical engineering, Computational science

## Abstract

Stent graft devices for the treatment of abdominal aortic aneurysms (AAAs) are being increasingly used worldwide. Yet, during modelling and optimization of these devices, as well as in clinical practice, vascular sections are idealized, possibly compromising the effectiveness of the intervention. In this study, we challenge the commonly used approximation of the circular cross-section of the aorta and identify the implications of this approximation to the mechanical assessment of stent grafts. Using computed tomography angiography (CTA) data from 258 AAA patients, the lumen of the aneurysmal neck was analysed. The cross-section of the aortic neck was found to be an independent variable, uncorrelated to other geometrical aspects of the region, and its shape was non-circular reaching elliptical ratios as low as 0.77. These results were used to design a finite element analysis (FEA) study for the assessment of a ring stent bundle deployed under a variety of aortic cross-sections. Results showed that the most common clinical approximations of the vascular cross-section can be a source of significant error when calculating the maximum stent strains (underestimated by up to 69%) and radial forces (overestimated by up to 13%). Nevertheless, a less frequently used average approximation was shown to yield satisfactory results (5% and 2% of divergence respectively).

## Introduction

Since 1991, when Parodi^1^ first reported endovascular aneurysm repair (EVAR), the implanting of a stent graft inside an abdominal aortic aneurysm (AAA), the procedure has become mainstream, with recent data ranking it the most common technique for repairing AAAs^2^.

When compared to open surgical repair, EVAR has shown to have lower short-term rates of death and complications^3^. This initial survival benefit, though, is lost a few years after the operation^3,4^, due to late medical complications. Moreover, EVAR is more expensive^4^ and leads to more readmissions^5^. Though it is true that, being a minimally invasive technique, EVAR is significantly more convenient for the patient (shorter operating time, less blood loss and shorter hospitalization), it has still to prove its long term superiority. Current judgment can be found in a recent review^6^ by the European Society of Vascular Surgeons, who present a considered and extensive set of guidelines for the management and treatment of AAA’s balancing the efficacy of both EVAR and OSR when required.

The most common complications of the EVAR procedure are endoleaks, occurring when the aneurysm is not completely excluded from the circulation and device migration, caused by a loss of structural integrity between the endograft and the vessel. Endoleak occurrences range in the literature from 10% to 45%^7^ while migration incidents have been reported to be as frequent as 19%^8^. In general, within 4 years post EVAR, it is estimated that 40% of patients will experience some form of a device-related complication and half of them will undergo a secondary intervention^7^. Inadequate anchoring of the device and a decrease in radial forces exerted from the endograft are usually the main reasons promoting these effects^9,10^.

Endoleaks, migration and stent mechanical failure are all dependent on the endograft design as well as the way it couples with the vascular wall^9,11,12^. While the causes of endoleaks can be attributed to graft permeability and long-term vascular dilation we focus on one of the key requirements for graft sealing; the mechanical interaction of stent graft and the artery wall and specifically the forces acting at the stent-artery interface, responsible for endoleak type I. For that, finite element analysis (FEA) models have been extensively used in the literature, thoroughly examining and optimizing mechanical aspects of medical significance.

In most of these studies, vessels are treated as idealized tubes with a circular cross-section, yet the assumption of circularity is challenged when examining CTA scans of the aorta^13–15^. It is indeed true that in clinical practice, medical doctors consider the aortic cross section to be circular and its radius is usually approximated either with the minimum radius^16^ or the maximum radius^17,18^ appearing on the medical imaging data. Yet the consequences of this practice have not been evaluated in the EVAR context.

Almost every investigation of the mechanical response of stent deployment is carried out assuming circular boundary geometries (for some recent examples refer to^19–22^). Even where analysis of plaque is attempted^23–25^ or patient specificness in taken into account^26^, the assumption of a circular luminal cross-section is usually maintained. Recognising the need to move away from such idealizations, some researchers^27^ have proposed the development of tools that can take into account all geometrical irregularities of vessels, while others have developed such algorithms^28^ when reconstructing the aortic geometry from 2D data. However, to the authors’ knowledge, no thorough investigation has ever been conducted to quantify either the cross-sectional shape variations of the human abdominal aorta or its effects on stent graft deployment.

Herein, the circularity assumption is challenged by investigating the cross-section of the neck of the AAA. The significance of this region to the EVAR is critical since it directly affects the proximal end of the stent graft, which is primarily responsible for preventing endoleaks and migration. The effects of the vascular cross-section on the exerted forces and strains of the stent are also examined via FEA, because of their importance to sealing and fatigue life.

## Results

### CTA Study

**Figure 1:**
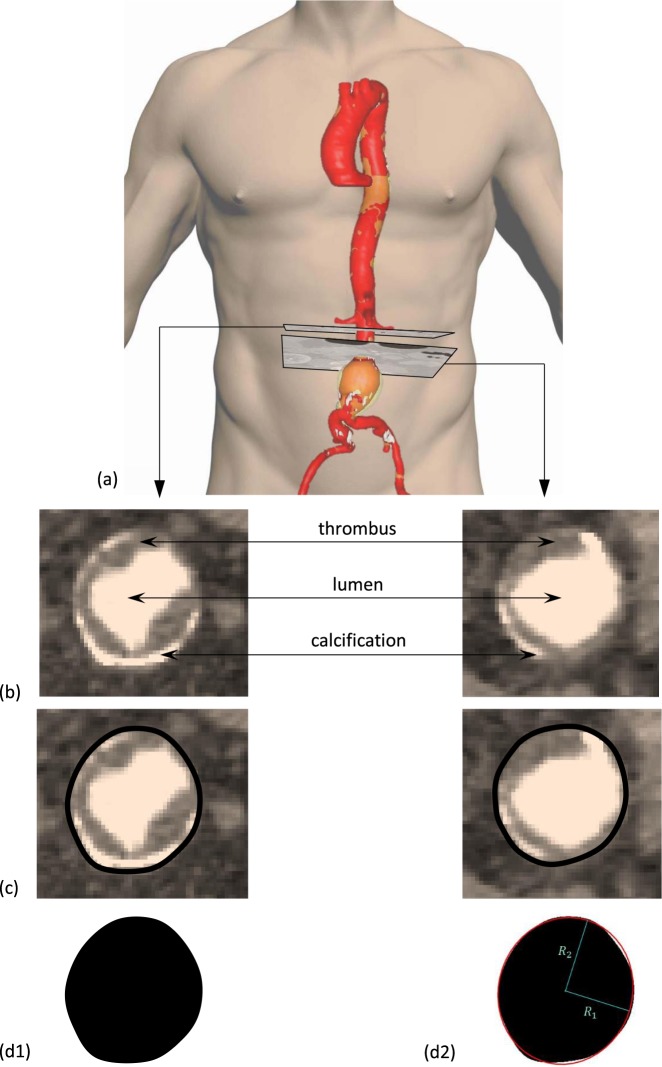
For every patient, 2 CTA slices were examined, one at the proximal (left column) and one at the distal end (right column) of the aneurysmal neck. For each image (**b**) a border on the intima was manually drawn (**c**) and then turned into a binary image (d1). Figure (d2) shows the distal cross-section of a patient with the equivalent ellipse superimposed on top of it, allowing the definition of $\,ER$. Thrombus and calcification (transparent yellow and white regions in (**a**)) were included in the cross-section. Source of the image of the torso: GermanVectorPro/Shutterstock.com.

**Figure 2:**
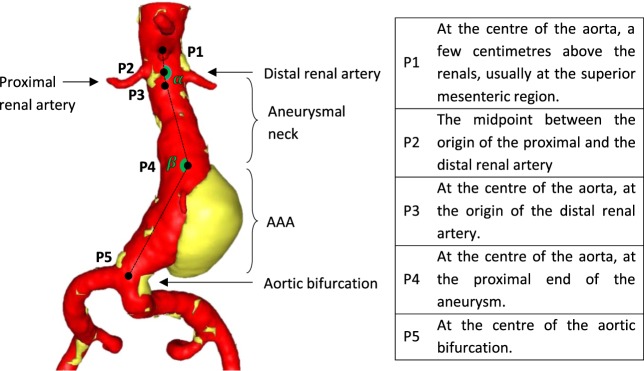
Dimensional and angular variables related to the AAA neck.

We examined pre-op computed tomography angiography (CTA) scans from 258 AAA patients that subsequently underwent EVAR. For each patient, a proximal and a distal image of the proximal AAA neck was acquired and the luminal shape was extracted (Fig. 1). In each cross-section, the circularity factor ($CF$) and elliptical ratio ($ER$) were used as metrics of shape. The results are summarised in Table 1. Furthermore, using 5 points of interest per case (Fig. 2), an additional four variables related to the aneurysmal neck, were examined. Table 2 reports the median and range of them, while histograms of all examined variables are provided as Supplementary Information.

**Table 1:** The median and extreme values of the metrics quantifying the shape of the AAA’s neck.

Variable	Median	Min	Max
Average $CF$	0.996	0.98	1.00
Average $ER$	0.940	0.83	0.99
$CF$	—	0.97	1.00
$ER$	—	0.77	1.00

The first two rows result from the averaging of the proximal and distal cross-sections while the bottom two refer to any cross-section of the dataset.

**Table 2:** The median and range of geometrical variables related to the AAA neck.

Variable	Median	Range
**Average Neck Diameter [mm]** *The mean value of two neck diameters, one at the distal renal artery (P3) and one at the most distal extent of the proximal neck (P4)*	23.1	16.8–34.2
**Neck Length [mm]** *Centreline distance of the AAA neck (path length between points P3 and P4)*	22.0	2.0–50.0
**Angle** ${\boldsymbol{\alpha }}$ **(in 3D space) [deg]** *from the triad of points (P1, P2, P4)*	159	68–179
**Angle** ${\boldsymbol{\beta }}$ **(in 3D space) [deg]** *from the triad of points (P2, P4, P5)*	120	31–177

Average $CF$ and average $ER$ were compared to each other. The metrics had a Pearson’s correlation value of 0.684, suggesting that the shape factors are producing similar values to each other, hence no significantly rough boundaries were present in the intima. More interestingly, neither of the two metrics strongly correlated to any of the additional variables of the aneurysmal neck and only $CF$ presented a very weak correlation of 0.161 with angle $\,\alpha$.

When comparing the values of the metrics on the two planes of the cross-section of the aortic neck (i.e. the relation between the proximal and the distal cross-section), only a weak correlation appears (ρ = 0.237 for $CF$ and ρ = 0.206 for $\,ER$), suggesting that, if possible, the study of two cross-sections in the aortic neck’s region should be pursued.

Neither $ER$ nor $CF$ was found to follow a normal distribution, an expected result since both metrics have an upper limit of 1. Furthermore, no shape metric had a statistically significant difference between the genders, despite differences in the neck length, which was statistically significantly bigger in males (23.0 mm vs 16.0 mm for females), as well as the average neck diameter (23.5 mm vs 21.25 mm).

**Figure 3:**
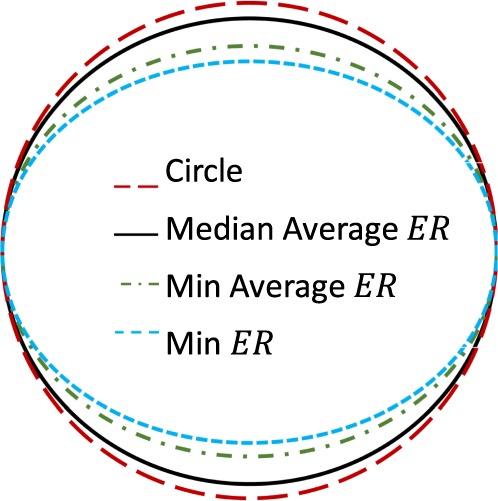
Schematic visualization of $ER$ results. ‘Average’ corresponds to the averaging of the proximal and distal site results.

In general, $CF$ was high, suggesting the smoothness of the intima. And while the median average $ER$ was also high (closely resembling a circle), in instances, $ER$ got as low as 0.77 (Fig. 3). This highlights the need for special patient specific attention when designing and modelling stent grafts, since the AAA neck is the most proximal region of endograft contact and is primarily responsible for adequate EVAR anchoring and sealing. The effect of such a cross-section on the deployment of an endograft was subsequently examined.

### FEA deployment study

**Figure 4:**
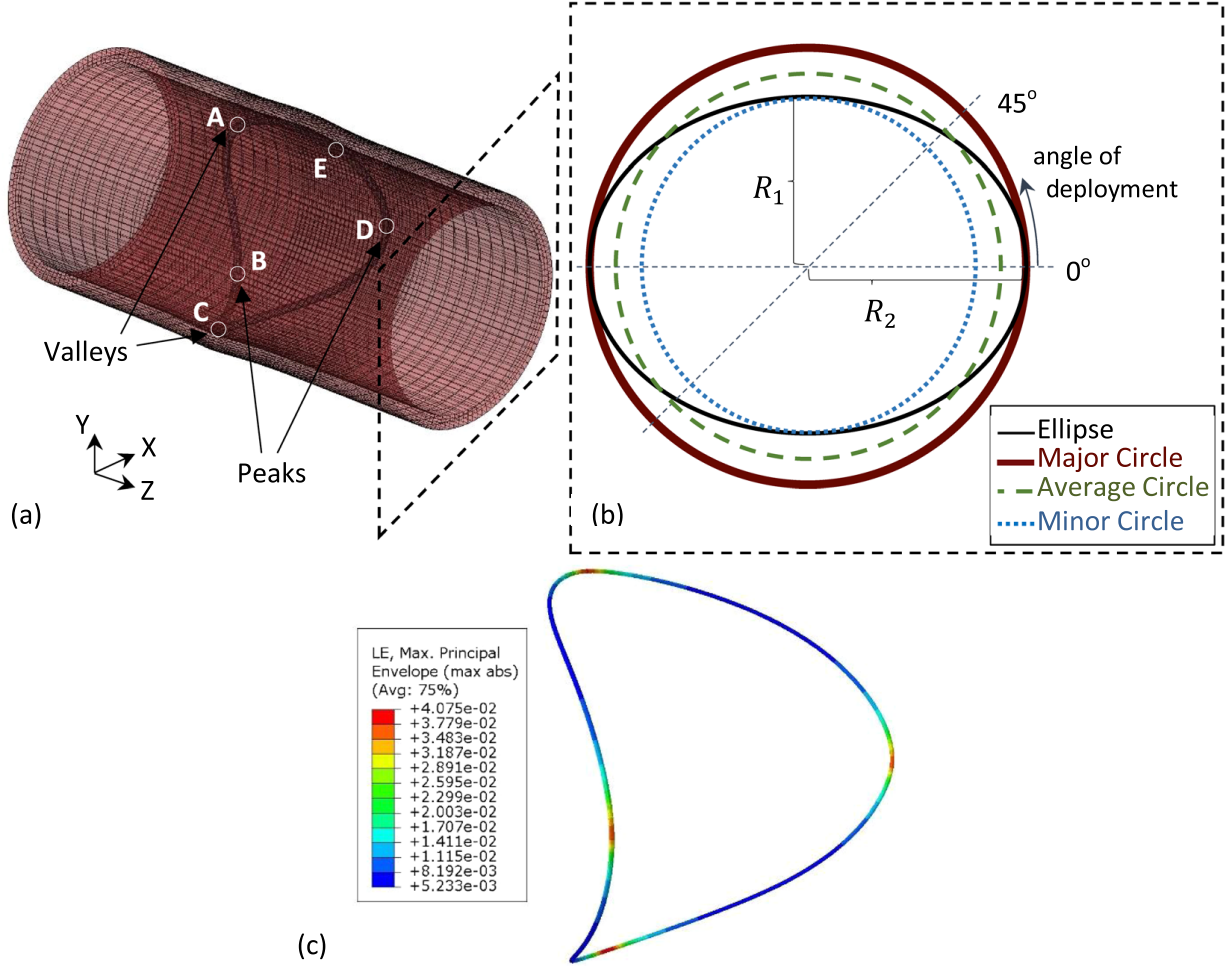
The ring stent bundle and vessel model used for the FEA study (**a**) and a schematic of the vascular cross-section (**b**). The elliptical ratio changed from 1 (brown circle) up to 0.75 (black ellipse) at the pressurized state. For every one of the ellipses, a circle with diameter equal to its minor axis (blue dotted circle) and an average diameter circle (green dashed circle) were examined. Additionally, 3 angles of deployment were studied for each elliptical section. In (**c**), the logarithmic strains of the ring deployed in the most extreme elliptical vessel are visualized.

A FEA model was used to explore the effects non-circular cross-sections have on deployed stents (Fig. 4). A model of a ring stent bundle, found in devices like the Anaconda^TM^ (Terumo Aortic, Glasgow, UK), was deployed in a number of elliptical vessels, as well as complementary circular ones. All cross-sectional configurations are reported in Table 3: every ellipsis was associated with a minor, a major and an average circle, each of which corresponds to a different clinical approximation of the lumen (Fig. 4b). Note that the values of Table 3 correspond to the original build of the vessels, hence refer to the unpressurized state. Note also that the most extreme of these cases was designed so that when pressurized, the most severe $ER$ of the CTA study would be approximated.

**Table 3:** Cross-section specifications of the FEA vessel.

Elliptical Ratio	Ellipses		Circles	
	${{\boldsymbol{R}}}_{2}$	${{\boldsymbol{R}}}_{1}$	Minor Circle ${\boldsymbol{R}}$	Average Circle ${\boldsymbol{R}}$
1.00	9.98	9.98	9.98	9.98
0.94	9.98	9.38	9.38	9.68
0.89	9.98	8.88	8.88	9.43
0.83	9.98	8.28	8.28	9.13
0.77	9.98	7.68	7.68	8.83
0.71	9.98	7.09	7.09	8.53
0.65	9.98	6.49	6.49	8.23

Values are reported in mm at 0 mmHg (unpressurized state). The major circle has a constant radius of 9.98 mm.

**Figure 5:**
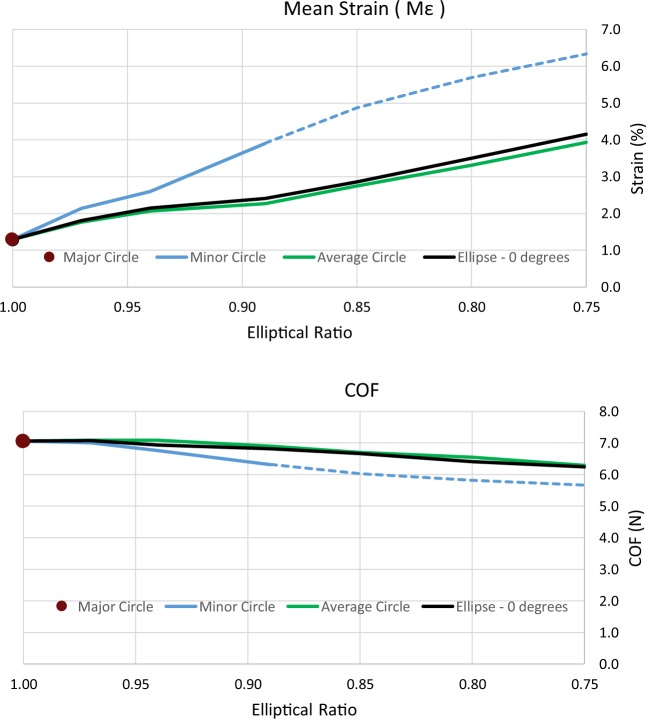
Maximum mean strain developed on the ring (top) and COF exerted by the ring at the end of systole (bottom) versus the elliptical ratio of the pressurized vessel. Ring deployed in all circles and the ellipses at 0° degrees. The dotted line refers to oversize greater than 33%.

In all models examined, the maximum strain of the ring stent bundle increased while the elliptical ratio decreased (Fig. 5 top). The worst case ellipse ($ER=0.75$) had a maximum strain of 0.0415 and if the major axis approximation was followed, the strain would be underestimated by 69%. At the average elliptical ratio ($ER=0.94$), this value dropped to 40%, still yielding a significant error. In contrast, the minor axis approximation would result in a 62% overestimation at $ER$ = 0.89 (elliptical ratio that results in 33% oversize) and 21% at $ER=0.94$. Regarding the average circle, an underestimation was also observed but it was significantly smaller: 5% at $ER=0.75$ and 3.5% at $ER=0.94$.

A smaller variation was observed for the chronic outward force (COF) (Fig. 5 bottom). The major axis approximation overestimated COF by 13.1% at the worst case ellipse and by 1.8% at the average ellipse. In contrast, the minor circle underestimated COF by 7.3% at the 0.89 ratio and by 2.5% at the 0.94 ratio. The average circle laid 0.8% above the extreme elliptical value and 2.2% above the average one.

**Figure 6:**
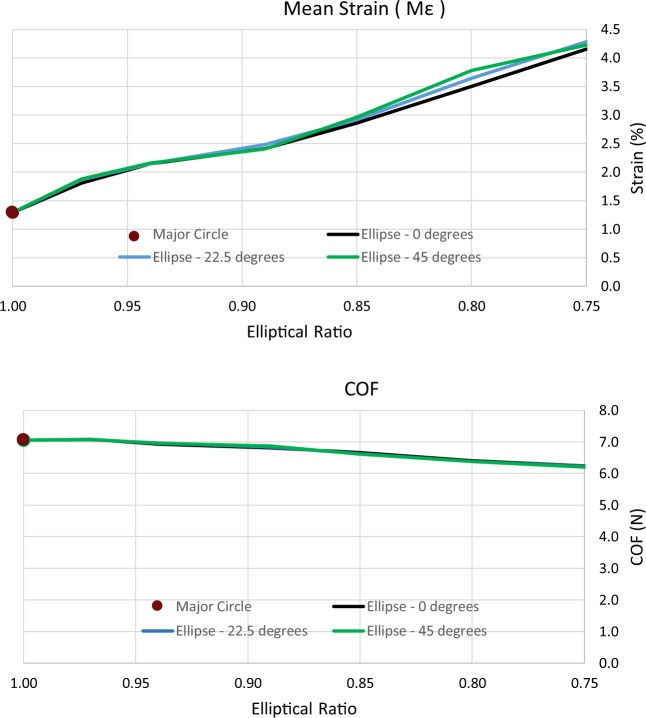
Maximum mean strain developed on the ring (top) and COF exerted by the ring at the end of systole (bottom) versus the elliptical ratio of the pressurized vessel. Ring deployed in the ellipses at various deployment angles.

When examining the differences that arise once the deployment angle of the endograft changes (angle of rotation about luminal axis), results are less sensitive. COF does not change more than 1% at any configuration (Fig. 6 bottom) while maximum strain stays below a 4% variation (Fig. 6 top) for most of the configurations.

**Figure 7:**
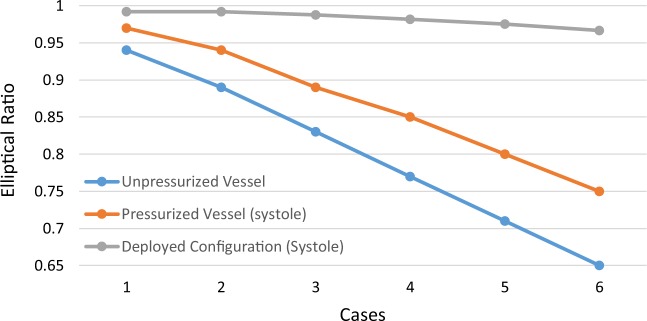
The effect of stenting on the ellipticity of the vessel.

Finally, it is interesting to note that similar to the effect the vessel has on the stent, the stent affects the cross section of the vessel as well. In all simulations, the post-deployment shape of the lumen was significantly altered by the bundle ring, hence conforming into a circular shape (Fig. 7). The most extreme cross section constructed had an *ER* = 0.65 when unpressurized: upon pressurization this ratio increased to 0.75 and after the ring deployment it was further raised to 0.966.

## Discussion

The cross-section of the AAA neck was extensively examined through the study of 258 AAA patients, challenging the assumption of circularity. Results showed that the median average $ER$ did not lie far from the circle, yet aneurysmal necks with very elliptical cross-sections were identified as well. Furthermore, the lack of correlation between the shape metrics used and other geometrical variables of the AAA neck, indicate that special attention needs to be taken for the aortic neck’s luminal shape in a patient by patient manner.

The scans used for vascular measurements should ideally be perpendicular to the centreline of the vessel. Nevertheless, axial scans are sometimes used in clinical practice^29^. In such cases, the smallest measurement of the luminal diameter is considered an appropriate approximation of the true diameter^30^, yet the errors introduced with this approximation are not minimal. It has been shown that the use of axial scans leads to statistically significant overestimations of the aneurysmal size when compared to scans transverse to the vessel^31^. Even if the scan orientation is perpendicular to the lumen though, when non-circular target vessels are identified, specialists make use of approximate diameters to plan the EVAR and decide upon the most suitable endograft. Nevertheless, as already mentioned, it is not rare that stents fail to anchor or seal appropriately, incidents that might be aided by non-circular aortic cross-sections. Utilizing the CTA study, a thorough examination was conducted exploring the effects the cross-sectional shape of the vessel has on a stent model and both the COF and (especially) the strains were found to be significantly affected.

The major axis approximation was found to underestimate the maximum strain by 69% at the most extreme $ER$ and the total radial force overestimated by 13%. In contrast, the minor axis approximation overestimated the maximum strain by up to 62% and underestimated the total radial force by up to 7%. These two approaches represent the most common approximation of the vascular cross-section and if followed, can be the source of significant errors during EVAR planning. On the other hand, the average circle produced a much better estimation of the maximum strain and overestimated the COF by a maximum of 2.2% when compared to the respective elliptical shapes. This is important, since the underestimation of strains can cause unexpected fatigue fractures and result in the loss of structural integrity of the device while the overestimation of radial forces may lead to unforeseen loss of stent/vessel sealing. As a result, the average circle can be safely used to calculate these variables, when a CT perpendicular to the aortic centreline is considered.

When examining the axial deployment angle of the endograft, it was demonstrated that the COF and maximum strains were practically unaffected by it. Furthermore, it was calculated that the post-deployment shape of the lumen, being strongly affected by the bundle ring, significantly increased its $\,ER$, diverging only by 3.4% from a circle even in the most extreme elliptical case.

The CT scans obtained for this study reflect a specific group of AAA patients, i.e. those who are suitable for EVAR. Nevertheless, given that the results of this analysis are relevant to EVAR interventions only, the nonrepresentation of the AAA population suitable for open repair is not considered a limitation. In the future, pre- and post-EVAR data should ideally be analysed in parallel, to examine if vascular non-circularity can be a predictor of endoleaks or migration. Despite this omission though, it is believed that the variables examined herein already improve our current understanding of the geometry of the aortic anatomy and bring new insight to the topic. It is recommended that future stent graft analyses take into account variations in the target vessel geometry, since the use of non-idealized cross-sections can have a measurable effect on the functional performance of these devices. It is also recommended that medical practitioners use average aortic diameters during EVAR planning. The accurate sizing of the endografts will result in optimized device functionality and reduced EVAR complications.

## Methods

### CTA study

The data used herein, were collected by the core laboratory M2S, Inc. West Lebanon, NH, USA in the period May 2009 to July 2011 as part of the non-randomized Anaconda stent graft investigational device exemption (IDE) pivotal study, approved by the U.S. Food and Drug Administration (registration number: NCT00612924 in the U.S. National Library of Medicine^32^) in accordance with relevant guidelines and regulations. All patients provided their informed consent. For the purposes of the current study, preoperative (baseline) imaging data from 258 patients (222 (86%) men, 34 (13%) women and 2 (1%) unspecified) aged between 51 and 88 years were used. Data were supplied to us anonymised, with age and gender being the only descriptors of the patients. The inclusion criteria of the original study were:

- Infrarenal AAA ≥ 4.0 cm in diameter, or AAA growth ≥1.0 cm/year
- Iliac artery distal fixation sites ≥20 mm in length
- Ability to preserve at least one internal iliac artery
- Femoral/Iliac artery’s size and morphology should be compatible with the appropriate delivery system (6 mm, 6.67 mm or 7.67 mm in diameter)

Patients with thrombus, calcification and/or plaque ≥2 mm in thickness and/or 50% continuous coverage of the vessel’s circumference in the intended fixation site were excluded. Pre-op data were supplied to us anonymised, with age and gender being the only descriptors of the patient and m2s Preview v4.0.1 software was used to assess them.

For our study, 2 CTA images were considered per patient, one at the proximal end of the AAA’s neck (immediately below the lower renal) and one at its distal end, right before the aneurysm (Fig. 1). All images were acquired at a plane perpendicular to the aortic centreline. This is important, since the original (axial) scans are not, in general, transverse to the axis of the vessel and can make cross-sections appear non-circular or asymmetric even if they are not, especially if significant tortuosity is present.

Inside most AAAs, there is usually intraluminal thrombus (ILT) and regions of calcification. The irregularity of ILT and calcification is vast and random, yet it is reasonable to assume that deployed stents crush ILT and part of the calcified layer, especially when ballooning is employed for this reason exactly. As a result, thrombus and calcification were not included in the current analysis.

For each CTA image, a cross-section boundary that included the lumen, the calcified and the thrombotic region was manually drawn. This allowed the definition of the intima wall’s inner border. The images of these borders alone were then manipulated with a Matlab script (version R2015b, MathWorks), turned into binary format and filled to create a 1-colour 2D surface. In each final surface two metrics were applied to quantify their divergence from circularity.

The first metric used was the circularity factor, defined as:

1 $$CF=4\pi \frac{A}{{P}^{2}}$$

where A = area and P = perimeter of the examined surface. This metric is affected by ovality as well as irregular (i.e. jagged or rough) edges and for a circle gets a maximum value of 1.

The second metric was the elliptical ratio and for the calculation of it, as a first step, the evaluation of an equivalent ellipse (same second moment of area as the original surface) was necessary. Given the ellipse, elliptical ratio is defined as:

2 $$ER=\frac{{R}_{1}}{{R}_{2}}$$

${\rm{where}}\,{\rm{R}}1={\rm{semi}}-{\rm{minor}}\,{\rm{axis}}\,{\rm{of}}\,{\rm{the}}\,{\rm{ellipse}}\,{\rm{and}}\,{R}_{2}$ *=* semi-major axis of the ellipse (Fig. 1d2).

By averaging the values of each metric for the proximal and the distal cross-section, average $CF$ and average $ER$ could be acquired to represent each AAA neck.

An additional 4 variables were considered in the region of the AAA’s neck, in order to reveal any possible correlations between the shape of the neck and its luminal outline. The Average Neck Diameter, calculated as the average value of two neck diameters, one at the distal renal artery (P3) and one right before the proximal end of the AAA (P4), the Neck Length, defined as the centreline distance between points P3 and P4 and the angles $a$ and $\,b$, calculated from the points (P1, P2, P4) and (P2, P4, P5) respectively, were examined (Fig. 2). Note that the measurements of the Average Neck Diameter and Neck Length were conducted by trained technicians of M2S, as part of the initial clinical investigation.

Finally, the shape metric data, along with the neck-related variables, were statistically analysed using SPSS, version 25. Normality was tested according to the Shapiro-Wilk test. Correlation between the examined variables was investigated with a 2-tailed Pearson correlation test at the 0.01 significance level. Lastly, the difference of medians between the genders was examined with the non-parametric median test for 2 independent medians at the 0.05 significance level.

### FEA deployment study

Utilizing the results of the CTA study, a numerical analysis of the non-circularity of the aorta was conducted. More specifically, a series of 19 elliptical and “equivalent-circle” vascular sections in the range of $ER$ values identified previously was considered as target vessels of EVAR. A FEA stent model was assembled, compacted and deployed in each vascular section, and mechanical assessment followed. The FEA stent model used had been previously developed and validated^33^; it is a ring stent bundle model, typical of the structural units found in the Anaconda^TM^ endograft. In brief, it consists of a series of straight overlapping Nitinol wires that curve into a ring shape, to take into account the strains induced by the manufacturing process. The material parameters of Nitinol and the ring specifications used herein are summarized in Tables 4 and 5 respectively.

**Table 4:** Parameters for the constitutive model of Nitinol.

Austenite elasticity $\,{E}_{A}$ (GPa)	59
Austenite Poisson’s ratio $\,{\nu }_{A}$	0.33
Martensite elasticity ${E}_{M}$ (GPa)	26.5
Martensite Poisson’s ratio ${\nu }_{M}$	0.33
Transformation strain ${\varepsilon }^{L}$ (MPa)	0.05
Start of transformation loading ${\sigma }_{L}^{S}$ (MPa)	636
End of transformation loading ${\sigma }_{L}^{E}$ (MPa)	740
Start of transformation unloading ${\sigma }_{U}^{S}$ (MPa)	430
End of transformation unloading ${{\rm{\sigma }}}_{{\rm{U}}}^{{\rm{E}}}$ (MPa)	302
Start of transformation stress in compression (MPa)	965

**Table 5:** The specifications of the FEA ring stent bundle model.

Variables	Ring
Wire Diameter [mm]	0.180
Ring Mean Diameter [mm]	27.02
Number of Turns	10
Bundle Diameter [mm]	0.69

The neck of the aneurysm was represented by a straight tube with length twice the ring diameter and original thickness of 1.9 mm. By altering the semi-minor axis, ${R}_{1}$, of the cross-section, the elliptical ratio was adjusted according to Eq. (2) (Fig. 4).

Starting from the value of 1, representing a circle, 6 more equidistant ratios were examined down to the value of 0.65, which when pressurized increased to 0.75, i.e. close to *ER* = 0.77, the most extreme ratio identified in the patient dataset (Table 3). Taking into account the geometrical shape of the ring, for each ellipse, 3 cases were studied, each one considering a different deployment position (Fig. 4b): (i) with the peaks and valleys of the ring being aligned with the axes of the cross-section, (ii) with the ring peaks at 22.5° angle and (iii) with the ring peaks at 45°. For symmetry reasons, the 0° and 45° are the extreme cases of rotation about the axial direction of the vessel. Additionally, two more cases were examined for each ratio different to 1: a circular cross-section with radius equal to ${R}_{1}$, called minor circle, and a circular cross-section with radius $\frac{{R}_{1}+{R}_{2}}{2}$, called average circle.

The reason for examining the circular cases was to compare the ellipses with the results that would occur if common circular approximations were followed. The original circle represents the case of approximating the ellipse with its major axis, the minor circle corresponds to the minor axis approximation and the average circle lies in between.

In each vascular section, the ring model was deployed assuming a friction coefficient $\mu$ = 0.3. The vessel was pressurised between diastole (${P}_{d}$ = 80 mmHg) and systole (${P}_{s}$ = 160 mmHg) for 5 cycles that allowed the system to settle. The maximum mean strain developed on the ring between systole and diastole was exported along with the COF (calculated by summing the radial component of all forces produced by the ring on the vascular surface) at the end of the systolic phase. The boundary conditions applied to the ring are summarized below (refer to Fig. 4a, for point definitions).

During compaction:

3 $${{\rm{Rotation}}}_{{\rm{x}}}=0\,{\rm{for}}\,{\rm{points}}\,{\rm{B}},{\rm{D}}$$

4 $${{\rm{Rotation}}}_{{\rm{y}}}=0\,{\rm{for}}\,{\rm{points}}\,{\rm{A}},{\rm{C}}$$

5 $${{\rm{Rotation}}}_{{\rm{z}}}=0\,{\rm{for}}\,{\rm{points}}\,{\rm{A}},{\rm{B}},{\rm{C}},{\rm{D}}$$

6 $${{\rm{Displacement}}}_{{\rm{x}}}=0\,{\rm{for}}\,{\rm{points}}\,{\rm{A}},{\rm{C}}$$

7 $${{\rm{Displacement}}}_{{\rm{y}}}=0\,{\rm{for}}\,{\rm{points}}\,{\rm{B}},{\rm{D}}$$

8 $${{\rm{Displacement}}}_{{\rm{z}}}=0\,{\rm{for}}\,{\rm{points}}\,{\rm{B}},{\rm{D}}$$

During deployment:

Equations (3)–(5)

9 $${{\rm{Displacement}}}_{{\rm{z}}}=0\,{\rm{for}}\,{\rm{point}}\,{\rm{B}}$$

During pressurization:

Equations (3)–(5)

10 $${{\rm{Rotation}}}_{{\rm{y}}}=0\,{\rm{for}}\,{\rm{point}}\,{\rm{B}}$$

11 $${{\rm{Displacement}}}_{{\rm{z}}}=0\,{\rm{for}}\,{\rm{point}}\,{\rm{E}}$$

A 10% oversize (percentage by which a stent is larger than the target vessel) at the time-weighted arterial blood pressure *P*_*m*_ = 106.7 mmHg, defined as:

12 $${P}_{m}={P}_{d}+\frac{1}{3}({P}_{s}-{P}_{d})$$

was initially assumed. This oversize relates only to the initial circular vessel (major circle). Since the same ring was used for all vessel models, greater oversize ratios were acquired for all cases but the first. For the smallest average circle, oversize was 33% while for the smallest minor circle 69%. Nevertheless, because it is highly unlikely that a surgeon would plan an EVAR at 69% oversize, a maximum of 33% oversize was assumed for the minor circle as well. Hence, results above this region were not taken into account in post-processing and were only illustrated with dotted lines in the figures created. It should also be noted that when altering the radius of the vessel, the thickness of the vessel was altered too, so that each aortic section exhibited the same stiffness.

The material model of the vessel used was linear elastic and homogeneous. The material parameters are reported in Table 6 and were adjusted to produce a 4.2% radial strain on the vessel when stented, representing an extreme pulsatility scenario. As already mentioned, no thrombus or calcification were taken into account because of their versatile manifestation, the avoidance of such regions during EVAR planning, the fact that for the most part they get crushed during EVAR deployment, and the surprising range of stiffness values found in the literature (the elastic modulus for calcification ranges from 40 MPa^34^ up to 22.7 GPa^35^) making their accurate modelling questionable.

**Table 6:** Material parameters of the vascular section.

Elastic modulus [MPa]	1.65
Poisson’s ratio	0.49
Density [$tonne/m{m}^{3}$]	1.16·10^−9^

Finally, all simulations were run on Abaqus (version 6.13–2, Dassault Systemes Simulia Corp., RI, USA), on 12 Xeon^©^ CPUs of a desktop computer (3.40 GHz, 64 GB) and the analysis time was between 83 to 290 minutes, depending on the vessel size.

## Supplementary information

Supplementary information.

## Data Availability

All datasets associated with this manuscript are available in FIGSHARE (10.6084/m9.figshare.11742792) and from the corresponding author upon reasonable request.
