# Supplementary material for: Analysing The Cross-Section of The Abdominal Aortic Aneurysm Neck and Its Effects on Stent Deployment
Source: Sci Rep. 2020 Mar 13;10:4673. doi: 10.1038/s41598-020-61578-y (PMC7070033; doi:10.1038/s41598-020-61578-y)
Supplement: Supplementary file 1 — Supplementary information. [file 41598_2020_61578_MOESM1_ESM.pdf]

ANALYSING THE CROSS-SECTION OF THE ABDOMINAL AORTIC ANEURYSM NECK AND ITS EFFECTS ON STENT DEPLOYMENT

Faidon Kyriakou, William Dempster, David Nash

Supplementary information

Statistical distribution of the AAA proximal neck variables

The statistical distribution of all the variables examined are documented below. Results concern the analysis of 258 AAA patients enrolled in the 2009-2011 study: "Vascutek Anaconda stent graft system phase II IDE study". For variable definitions refer to Tables 1 – 2.

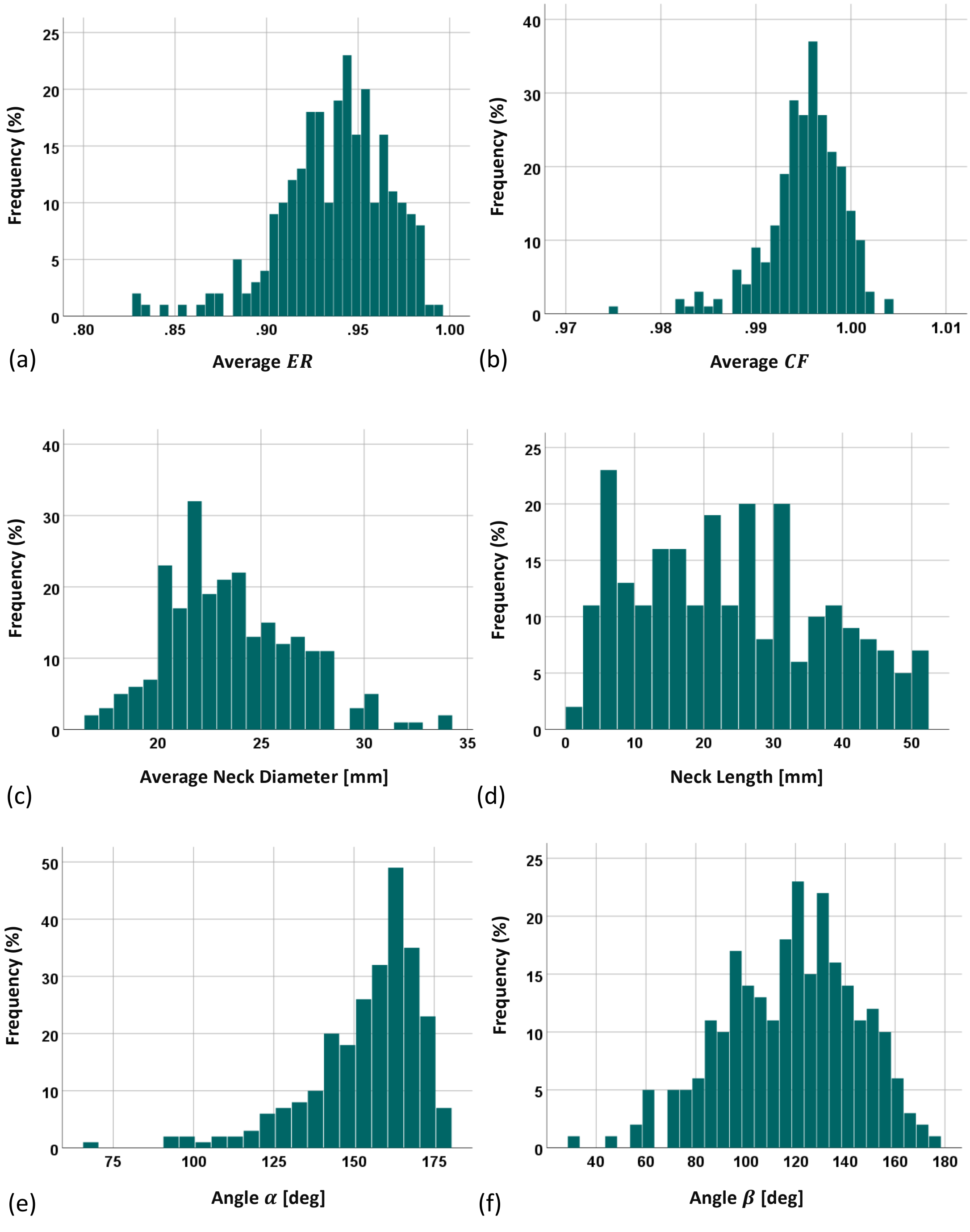

Fig. S1 Frequency distribution of the average elliptical ratio (a), average circularity factor (b), average neck diameter (c), neck length (d), angle α (e) and angle β (f).
